# Epidemiological characteristics analysis of childhood influenza A virus infections in Hebei, China (2020–2024)

**DOI:** 10.3389/fped.2026.1796772

**Published:** 2026-07-22

**Authors:** Yishuo Sun, Mingzhe He, Chan Wen, Han Wang, Ling Sun, Xiaodong Wang, Xiaowei Cui

**Affiliations:** 1 Department of Medical, Hebei Province of Children's Hospital, Shijiazhuang, Hebei, China; 2 Department of Orthopedics, Hebei Province of Children's Hospital, Shijiazhuang, Hebei, China; 3 Department of Hospital Infection, Hebei Province of Children's Hospital, Shijiazhuang, Hebei, China; 4 Intensive Care Unit (ICU), Hebei Province of Children's Hospital, Shijiazhuang, Hebei, China; 5 Hebei Provincial Clinical Research Center for Child Health and Disease, Shijiazhuang, Hebei, China

**Keywords:** children, China, epidemiology, Hebei, infection characteristics, influenza A virus

## Abstract

**Objective:**

To analyze the epidemiological and infection characteristics of childhood influenza A virus infections in Hebei, China, from 2020 to 2024, and to provide a basis for influenza prevention and control among children.

**Methods:**

A retrospective analysis was conducted on epidemiological and clinical data of children with influenza A virus infection who were hospitalized in Hebei Province from January 2020 to December 2024. Clinical specimens were analyzed using nucleic acid extraction reagents with automatic extraction on the Smart LabAssist-32 nucleic acid extractor. A 13-pathogen multiplex PCR detection kit combined with the GeXP multiplex gene expression analysis system was used for testing.

**Results:**

A total of 298,810 specimens from children with upper and lower respiratory tract infections were collected. Among which 60,928 specimens were positive for influenza A virus (detection rate: 20.39%). The infection rate was highest in 2024 (23.03%) and lowest in 2020 (13.57%). The annual positive detection rates of influenza A virus in Hebei from 2020 to 2024 were 13.57%, 16.97%, 19.95%, 20.41%, and 23.03%, respectively, with statistically significant differences (*P* < 0.05). The positive detection rate was significantly correlated with seasons, peaking in winter (29.04%) and reaching the lowest in spring and summer (5.4%). Mixed infections were detected in 16,049 specimens (26.34%), mainly involving rhinovirus, respiratory syncytial virus (RSV), and *Mycoplasma pneumoniae* (MP).

**Conclusion:**

Pediatric influenza A virus infections in Hebei Province exhibit a clustered age distribution, pronounced seasonal variation, and a high co-infection rate. Enhanced surveillance and protective measures should be targeted toward children aged 1–5 years and prioritized during the winter season. This study provides important scientific evidence for optimizing influenza prevention and control strategies, clinical diagnosis, and treatment.

## Introduction

Influenza A virus is one of the main pathogens causing seasonal influenza, with high variability and multiple subtypes. During influenza outbreaks in children, the incidence rate of influenza A can reach 30%–50% (1). Studies have reported that unvaccinated children are more susceptible to influenza infection and transmission (2). Typical clinical manifestations in children with influenza A include fever and cough, which may advance to severe pneumonia in severe cases. Children constitute a vulnerable population for critical influenza A disease, which may induce severe neurological complications such as acute necrotizing encephalopathy—a complication with a mortality rate exceeding 30% (3–5). Analysis of hospitalized pediatric patients with influenza A across Hebei Province reveals a clustered age distribution, prominent seasonal fluctuations, and a substantial co-infection burden. Targeted strengthened surveillance and protective interventions should be prioritized for children aged 1–5 years and fully implemented during winter epidemic seasons. Additionally, influenza A may be complicated by other bacteria and viruses, such as rhinovirus, adenovirus, and *Mycoplasma pneumoniae* (MP). Rhinovirus, one of the most common respiratory pathogens, synergizes with influenza A to evade early host immunity. Human metapneumovirus (hMPV), respiratory syncytial virus (RSV), and coronaviruses (e.g., HCoV-229E) are also frequently co-detected. Influenza A virus exhibits distinct seasonal epidemiological characteristics, with higher detection rates in winter than in summer (6–9).

Due to immature immune systems and frequent contact in collective settings such as schools, children have a high incidence of influenza during epidemic seasons, which can be fatal in severe cases. In recent years, with the rapid development of molecular diagnostic and biological detection technologies, influenza virus monitoring has attracted significant attention. Understanding its epidemiological patterns and pathogenic characteristics provides a basis for preventing and controlling influenza infections in local children (10–12). Currently, no studies have explored the epidemiological and etiological characteristics of childhood influenza A in Hebei Province. This study conducted a retrospective analysis of influenza virus detection data from hospitalized children with respiratory tract infections in Hebei Children's Hospital over five consecutive years (2020–2024), aiming to provide evidence for formulating diagnosis and prevention strategies for childhood respiratory tract infections in Hebei.

## Materials and methods

### Study subjects

Data were retrospectively collected from children under 18 years old hospitalized in Hebei Children's Hospital due to respiratory tract infections from January 2020 to December 2024, covering all counties and cities in Hebei Province. This study was approved by the Medical Research Ethics Committee of Hebei Children's Hospital (Ethics Approval No. 183).

The inclusion criteria were cases testing positive for influenza A virus and diagnosed with influenza A infection. A standardized case data collection form was developed to record all pediatric patient information, and a corresponding case database was established. Cases with incomplete data, children infected with COVID-19, and those with underlying diseases were excluded. Included cases were hospitalized children with positive detection in respiratory secretion etiological tests, diagnosed in accordance with the diagnostic criteria for respiratory tract infections (upper and lower respiratory tract) Acute upper respiratory tract infection refers to a general term for acute inflammation involving the nasal cavity, pharynx or larynx (13). The common cold, rhinitis, acute laryngopharyngitis, acute pharyngitis and acute tonsillitis are collectively categorized as upper respiratory tract infections. Acute lower respiratory tract infection is defined as the general designation of acute inflammatory lesions occurring in the respiratory tract below the larynx, including the trachea, bronchi, bronchioles and alveoli (13).

### Specimen collection

Specimen types included nasopharyngeal swabs, sputum, and bronchoalveolar lavage fluid. All respiratory specimens were collected within 24 h after hospital admission.

### Multiplex nucleic acid detection of respiratory pathogens

Thirteen respiratory pathogens were simultaneously detected, including influenza A virus, influenza A H1N1, influenza A H3N2, parainfluenza virus, influenza B virus, human metapneumovirus, RSV, adenovirus, rhinovirus, human bocavirus, human coronavirus, MP and *Chlamydia trachomatis*.

### Specimen processing

All respiratory specimens were processed within 2 h of collection. For nasopharyngeal swabs, swab tips were immersed in 2 mL of viral transport medium (VTM) and fully eluted by vortexing for 30 s. Sputum specimens were liquefied with 0.1% dithiothreitol (DTT) at room temperature before processing. A total of 200 μL of specimen supernatant was used for subsequent nucleic acid extraction.

### Nucleic acid extraction

Nucleic acid extraction was automatically performed using the Smart LabAssist-32 nucleic acid extractor (Dot Biotechnology Co., Ltd., Taipei, Taiwan, China) with matching extraction kits (Ningbo Health Gene Technology Co., Ltd., Ningbo, Zhejiang, China), in strict accordance with the manufacturer's operating manuals. Total nucleic acid (including viral RNA and bacterial DNA) was extracted via four standardized procedures: cell lysis, nucleic acid binding, impurity washing, and elution, with a final elution volume of 50 μL. Extracted nucleic acid samples were stored at −80 °C until further testing.

### Reverse transcription and multiplex PCR amplification

Subsequently, a commercial 13-pathogen multiplex PCR detection kit (Ningbo Health Gene Technology Co., Ltd., Ningbo, Zhejiang, China) coupled with the GeXP multiplex gene expression analysis system (Beckman Coulter Inc., Brea, California, USA) was applied for pathogen identification. The total 25 μL reaction system contained 12.5 μL of 2× reaction mix, 2 μL of enzyme mix (including M-MLV reverse transcriptase and hot-start Taq DNA polymerase), 5.5 μL of nuclease-free water, and 5 μL of extracted nucleic acid template. The thermal cycling procedure was set as follows: reverse transcription at 50 °C for 15 min; initial denaturation at 95 °C for 2 min; followed by 40 cycles of 94 °C for 30 s, 55 °C for 30 s, and 72 °C for 60 s; and a final extension at 72 °C for 5 min.

### Product detection and result interpretation

Amplified PCR products were detected and analyzed via capillary electrophoresis using the GeXP multiplex gene expression analysis system. The fragment size of each amplification product was compared with the reference standard provided in the kit to identify corresponding pathogens. A specimen was defined as positive for a specific pathogen when the peak height of the corresponding product exceeded the manufacturer's threshold, and the internal reference (human β-actin gene) showed a valid positive peak to confirm adequate specimen quality and experimental procedure validity.

### Statistical analysis

SPSS 24.0 software was used for database establishment, management, and statistical analysis. The chi-square test was used to compare constituent ratios or positive detection rates between groups. The significance level α was set at 0.05, and *P* < 0.05 was considered statistically significant.

## Results

### Baseline demographic characteristics of children with influenza A virus infection

A total of 298,810 hospitalized children presented with upper or lower respiratory tract infection symptoms and received respiratory pathogen testing during the study period. Only 60,928 children who met all inclusion and exclusion criteria were finally included; the remaining cases were excluded mainly due to incomplete laboratory test data, missing clinical records, concurrent COVID-19 infection, or complicated underlying chronic diseases.

There were 33,719 male and 27,209 female children, with a male-to-female ratio of approximately 1.24:1. Male children (55.34%) were more common than female children (44.66%) (Table 1). The youngest infected child was 44 days old, and the oldest was 15 years old. Statistical data showed that children aged 1–5 years constituted the largest proportion of children with influenza A virus infection; 14,114 children (23.16%) were younger than 1 year old, 29,147 children (47.84%) were aged 1–5 years, 17,369 children (28.51%) were aged 5–14 years, and only 298 children (0.49%) were ≥14 years old (Tables 2, 3).

**Table 1:** Gender distribution of hospitalized children with influenza A virus infection (2020–2024).

Year	Male	Proportion	Female	Proportion
2020	2,765	61.21%	1,752	38.79%
2021	3,340	59.72%	2,253	40.28%
2022	4,124	57.27%	3,077	42.73%
2023	7,378	57.83%	5,381	42.17%
2024	16,112	52.21%	14,746	47.79%
Total	33,719	55.34%	27,209	44.66%

**Table 2:** Age distribution of hospitalized children with influenza A virus infection (2020–2024).

Age	2020	2021	2022	2023	2024	Total	Proportion
<1	1,623	1,444	1,572	2,360	7,115	14,114	23.16%
1∼<5	2,248	3,236	3,321	5,428	14,914	29,147	47.84%
5∼<14	638	908	2,284	4,899	8,640	17,369	28.51%
≥14	8	5	24	72	189	298	0.49%
Total	4,517	5,593	7,201	12,759	30,858	60,928	100.00%

1∼<5 years refers to age ≥1 year and <5 years; 5∼<14 years refers to age ≥5 years and <14 years.

**Table 3:** Children of different age groups infected with influenza A virus.

Age	<1	1∼<5	5∼<14	≥14	*Χ* ^2^	*P*
Positive	14,114^a^	29,147^b^	17,369^c^	298^d^	22,616.97	<0.001
Tested Specimens	134,801	93,869	54,250	15,890		

Comparison among the four groups: *χ*^2^ = 22,616.97, *P* < 0.001. Different letter labels indicate statistically significant differences between two groups. A *P* value less than 0.05 was considered statistically significant.

### Annual positive detection rates of influenza A virus (2020–2024)

**Figure 1:**
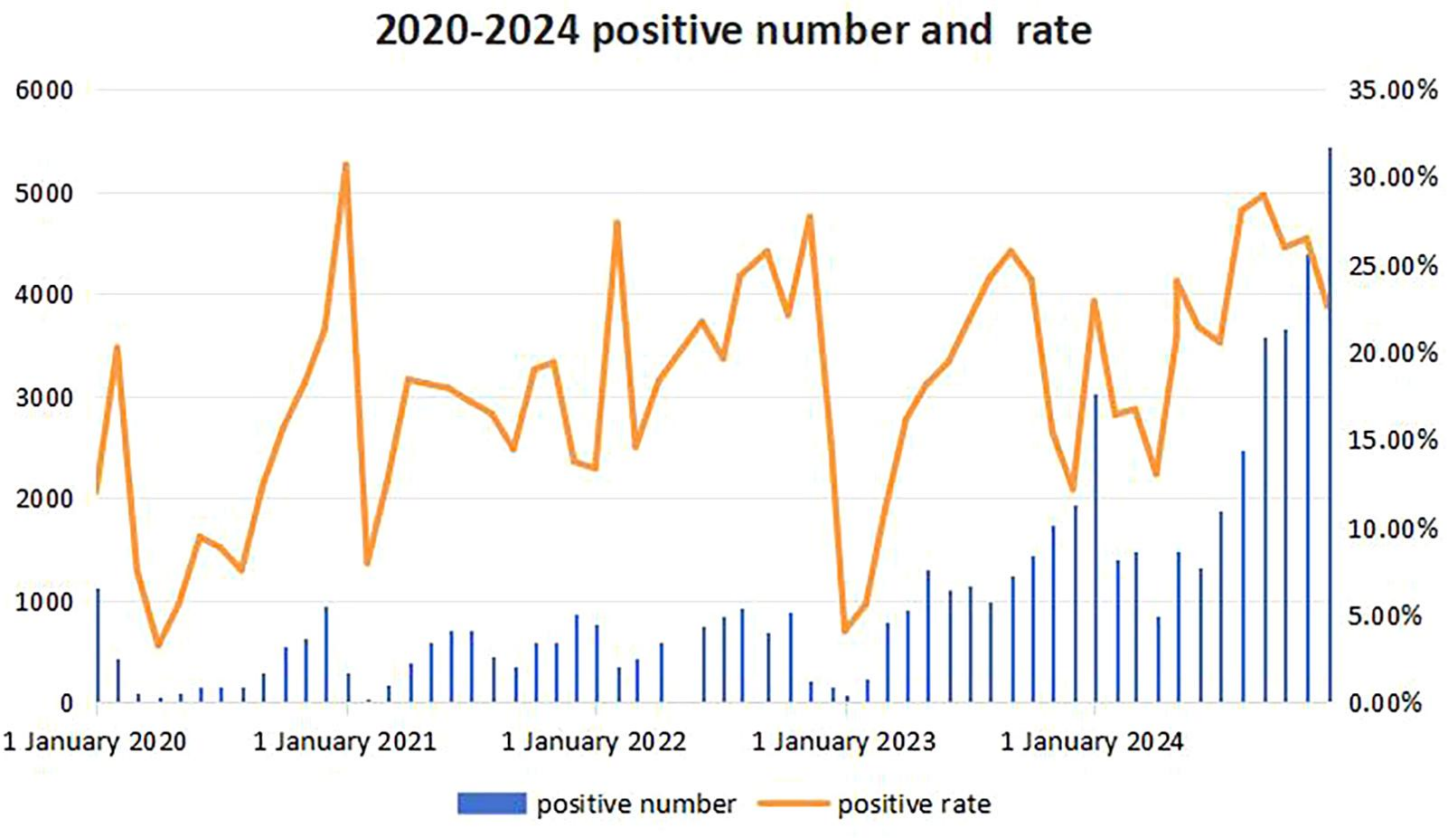
Seasonal distribution of influenza A virus positive detection rates (2020–2024). Spring: March, April, May; Summer: June, July, August; Autumn: September, October, November; Winter: December, January, February.

Children who tested positive for influenza A virus were identified in every study year. In total, 60,928 children with influenza A virus infection were included; 4,517 (13.57%), 5,593 (16.97%), 7,201 (19.95%), 12,759 (20.41%), and 30,858 (23.03%) children were infected with influenza A virus from 2020 to 2024, respectively. The positive detection rates were significantly lower than the 23.03% in 2024 (*χ*^2^ = 1772.1, *P* < 0.001) (Table 4). The peak season for influenza A virus infection occurred in winter (December, January, and February) each year, while the lowest positive detection rate was in summer, with the lowest in 2020 (Figure 1).

**Table 4:** Detection of influenza A virus-positive specimens (2020–2024).

Year	Positive	Tested specimens	Positive detection rate	*Χ*^2^, P*	*Χ* ^2^	*P* ^#^
2020_1_	4,517	33,281	13.57%	1,772.1 *p* < 0.001	1,431.1^1,5^	<0.001
2021_2_	5,593	32,961	16.97%		570.3^2,5^	<0.001
2022_3_	7,201	36,091	19.95%		155.5^3,5^	<0.001
2023_4_	12,759	62,513	20.41%		169.9^4,5^	<0.001
2024_5_	30,858	1,33,964	23.03%			
Total	60,928	2,98,810	20.39%			

For ease of understanding, footnotes are marked at the subscript of Year. The superscripts 1–5 denote the positive detection rates of different years. Chi-square tests were performed for comparisons among all five groups, followed by pairwise subgroup chi-square test comparisons. For instance, years 2020–2024 are categorized into Group 1, Group 2, Group 3, Group 4 and Group 5. The value *χ*^2^ = 1,431.1 (1,5) indicates the comparison between 2020 and 2024, while *χ*^2^ = 570.3 (2,5) represents the comparison between 2021 and 2024. P* indicates comparisons among the five groups, while *P*^#^ denotes separate comparisons with the year 2024.

### Mixed respiratory pathogen co-infection status

Among the 60,928 influenza A virus-positive respiratory specimens, co-infection with other respiratory pathogens was identified in 16,049 samples, corresponding to a mixed infection rate of 26.34%. Pathogens of mixed infections included rhinovirus, RSV, MP, metapneumovirus, adenovirus, and bocavirus. Cases with co-infection of three or more viruses accounted for 0.17%.

## Discussion

This study systematically analyzed hospitalized children with influenza A virus infection treated at a tertiary children's hospital in Hebei Province from 2020 to 2024, and described their epidemiological features by gender, age, seasonal distribution and co-infection status. These findings not only reflect the epidemiological patterns of children with influenza A virus infection in this region but also provide key data to support optimized prevention and control strategies, as well as clinical diagnosis and treatment plans.

This study found that among hospitalized children with influenza A virus infection from 2020 to 2024, male children (33,719 cases) outnumbered female children (27,209 cases), with a male-to-female ratio of approximately 1.24:1. This may be associated with innate immunity differences between males and females, which will be further explored in future studies. The proportion of male children was higher than that of female children in each year. This gender difference is consistent with the results of multiple domestic studies on childhood respiratory tract infections (14–17). The underlying causes of this difference are complex and result from the interaction of multiple factors. First, behavioral factors may play an important role: studies have shown that preschool and school-age boys are generally more active in outdoor activities, group games, and social interactions, which may increase their exposure to viruses. Second, gender differences in immune responses may be an intrinsic factor. Some studies suggest that during specific developmental stages, females may have stronger or earlier-maturing immune responses to certain viral infections, but the impact of this difference in childhood requires further confirmation. In addition, there may be gender biases in medical-seeking behavior; for example, families may be more concerned and alert to symptoms in boys, leading to higher hospitalization rates in statistics. However, this study is a single-center retrospective analysis and cannot deeply explore the underlying biological or sociological mechanisms. The exact cause of this difference needs to be clarified in future multi-center, prospective studies combined with detailed questionnaires and immunological tests.

Age analysis showed that children aged 1–5 years represented the highest-risk group for hospitalization related to influenza A virus infection, accounting for 47.84% of all children with influenza A virus infection, followed by infants younger than 1 year (23.16%) and children aged 5–14 years (28.51%). Adolescents aged ≥14 years accounted for an extremely low proportion (0.49%). This distribution highlights that infants and preschool children are high-risk groups for severe influenza. The main reasons are related to two aspects: first, the characteristics of immune system development. The active immune system of children aged 1–5 years is not yet fully mature, maternal antibody protection gradually fades, and immune memory against influenza A virus has not been fully established, resulting in relatively weak resistance to the virus. Infants under 1 year old, especially those under 6 months, have more fragile immune systems and are a high-risk group for complications. Second, environmental exposure factors: children aged 1–5 years begin to enter collective living environments such as nurseries and kindergartens, where dense populations and frequent contact create favorable conditions for the spread of respiratory viruses, greatly increasing the risk of infection. In contrast, the immune system function of adolescents aged ≥14 years has matured, and previous infections or vaccinations may have accumulated partial immunity. Most infections present as mild cases, and the need for hospitalization is significantly reduced. These results suggest that influenza prevention and control strategies and vaccine promotion should focus on children under 5 years old, especially those aged 1–5 years. It is recommended to vigorously promote influenza vaccination in this age group, strengthen health management such as morning checks and ventilation and disinfection in nurseries and kindergartens, and conduct health education for infant caregivers to form an effective protective barrier (18, 19).

This study found that the detection rate of influenza A virus in children in Hebei showed an overall upward trend from 2020 to 2024, increasing from 13.57% in 2020 to 23.03% in 2024, with statistically significant differences between years. This trend may be related to the superposition of multiple factors in recent years. The lowest detection rate in 2020 is considered to be closely related to the strict non-pharmaceutical interventions (such as universal mask-wearing, maintaining social distance, and school closures) implemented after the outbreak of the COVID-19 pandemic. These measures not only effectively curbed the spread of COVID-19 but also significantly inhibited the community transmission of influenza and other respiratory viruses. With the adjustment of epidemic prevention and control measures, the resumption of social activities, and the increase in population contact rates, the transmission conditions of influenza viruses have been restored, which may have led to a rebound in the infection base after 2021. This is consistent with the lack of natural immune stimulation in humans (immunity debt). In addition, the variability of the virus itself cannot be ignored. Influenza A virus is highly variable and may undergo antigenic drift, rendering partial immunity induced by previous infections or vaccinations ineffective, thereby leading to the accumulation of susceptible populations and an increase in infection rates (20, 21).

The seasonal distribution pattern observed in this study aligns with the typical epidemiological characteristics of pediatric influenza A in temperate regions. In Hebei, children with influenza A virus infection present a prominent winter epidemic peak, which is consistent with most domestic and foreign reports (14–17). The winter peak is the result of the interaction between climatic conditions, human behavior, and viral characteristics. First, cold and dry weather is conducive to the survival and stability of influenza viruses in the environment. Second, people spend more time indoors in winter, and closed doors and windows lead to poor ventilation, increasing the risk of aerosol and droplet transmission in confined spaces. Finally, collective units such as schools and kindergartens have a high degree of indoor aggregation in winter, which is prone to local outbreaks or epidemics. This distinct seasonal epidemic pattern provides a clear time window for implementing targeted prevention and control strategies, against a backdrop of year-on-year increases in viral detection rates. It is recommended to vigorously promote influenza vaccination in autumn (before the start of the epidemic season), strengthen the monitoring and early warning of influenza-like cases in winter and spring, and implement prevention and control measures in key places such as schools and hospitals.

Mixed infection is another focus of this study. Among the 60,928 specimens testing positive for influenza A, 16,049 samples presented co-infection with other respiratory pathogens, corresponding to a co-infection proportion of 26.34%. Rhinovirus, MP and RSV were the most frequent co-detected pathogens. This high proportion of mixed infections has important clinical significance. Mixed infections may lead to more complex clinical manifestations, such as prolonged fever, severe cough, and rapid progression of lung imaging, thereby increasing the risk of developing severe complications such as severe pneumonia and acute respiratory distress syndrome, and bringing challenges to clinical diagnosis and treatment. It reminds us that for children with severe clinical manifestations and poor response to conventional anti-influenza treatment, the possibility of co-infection should be highly vigilant. Clinically, molecular diagnostic technologies such as multiplex PCR should be actively used for broad-spectrum pathogen screening to achieve accurate diagnosis and avoid missed diagnoses. In terms of treatment, a comprehensive treatment plan including antiviral, antibacterial, or symptomatic support therapy should be formulated based on etiological results. In addition, the epidemiology and pathogenic mechanisms of co-infections with different pathogens, such as whether there is mutual promotion or inhibition between viruses, remain scientific issues worthy of in-depth study (20).

This study has certain limitations. First, it is a single-center retrospective study, and all data are from a provincial children's hospital. Its results may not fully represent the epidemiological characteristics of the general population in Hebei (including children treated in outpatient settings and children with community-acquired infection), and there is a certain selection bias. Second, the study is mainly based on laboratory test data and basic epidemiological information, lacking detailed clinical course, treatment plans, and prognosis data, so it is impossible to deeply analyze the specific impact of mixed infections on disease severity. In the future, multi-center, prospective cohort studies should be carried out, integrating detailed clinical, immunological, and virological data to more comprehensively reveal the disease burden and risk factors of childhood influenza.

## Conclusion

In summary, this study systematically characterizes the epidemiological features of hospitalized children with influenza A virus infection in Hebei from 2020 to 2024: more male children were affected than female children; children aged 1–5 years constituted the primary high-risk population; viral detection rates rose year by year, accompanied by a distinct winter epidemic peak; over one-quarter of pediatric patients experienced co-infections with other respiratory pathogens. Pediatric patients with influenza A in Hebei Province show a clustered age distribution, marked seasonal fluctuations, and a high co-infection prevalence. Strengthened surveillance and protective interventions should be targeted at children aged 1–5 years and prioritized throughout winter epidemic seasons. These findings provide important scientific evidence for formulating targeted influenza prevention and control strategies and optimizing clinical diagnosis and treatment. It is recommended to further promote childhood influenza vaccination, strengthen seasonal monitoring and health education, and reduce the incidence and severe risk of influenza (22–25).

## Data Availability

The datasets presented in this study can be found in online repositories. The names of the repository/repositories and accession number(s) can be found in the article/Supplementary Material.
